# Physicochemical Characterisation of Polysaccharide Films with Embedded Bioactive Substances

**DOI:** 10.3390/foods12244454

**Published:** 2023-12-12

**Authors:** Shweta Gautam, Lubomir Lapcik, Barbora Lapcikova, David Repka, Lilianna Szyk-Warszyńska

**Affiliations:** 1 Department of Foodstuff Technology, Faculty of Technology, Tomas Bata University in Zlín, Nam. T.G. Masaryka 5555, 760 01 Zlín, Czech Republic; gautam@utb.cz (S.G.); or barbora.lapcikova@upol.cz (B.L.); 2 Department of Physical Chemistry, Faculty of Science, Palacky University in Olomouc, 17. Listopadu 12, 771 46 Olomouc, Czech Republic; 3 Jerzy Haber Institute of Catalysis and Surface Chemistry, Polish Academy of Sciences, Niezapominajek 8, 30-239 Kraków, Poland; liliana.szyk-warszynska@ikifp.edu.pl

**Keywords:** crosslinking, encapsulation, carboxymethylcellulose, citric acid, edible films

## Abstract

In this study, sodium carboxymethyl cellulose (CMCNa) bioactive films, crosslinked with citric acid (CA), were prepared and comprehensively examined for their suitability in various applications, focusing on food packaging. The films displayed favourable properties, including appropriate thickness, transparency, and moisture content, essential for packaging purposes. Moreover, the films exhibited excellent moisture absorption rate and barrier properties, attributed to the high concentration of CMCNa and the inclusion of a CA. These films presented no significant effect of crosslinking and bioactive components on their mechanical strength, as evidenced by tensile strength and elongation at break values. Thermal stability was demonstrated in the distinct weight loss events at different temperature ranges, with crosslinking contributing to slightly enhanced thermal performance. Furthermore, the films showed varying antioxidant activity levels, influenced by temperature and the solubility of the films in different media, indicating their potential for diverse applications. Overall, these bioactive films showed promise as versatile materials with desirable properties for food packaging and related applications, where the controlled release of bioactive components is advantageous for enhancing the shelf life and safety of food products. These findings contribute to the growing research in biodegradable and functional food packaging materials.

## 1. Introduction

Edible films and coatings have a rich history that traces back to ancient China when people used lipid coatings to preserve fruits like lemons and oranges. However, in recent times, these edible packaging systems have undergone remarkable advancements, due to the need for more sustainable and environmentally friendly packaging solutions. Modern innovations have revolutionised edible films and coatings [1,2,3,4]. These biopolymeric packaging materials are designed to emulate the properties and functionalities of traditional packaging materials like plastic films and coatings [5,6,7,8]. They offer protection against potential factors like moisture and oxygen, safeguarding food quality. Additionally, the films can be used as carriers for functional ingredients, such as antimicrobial agents or antioxidants, further enhancing food preservation. One of the most significant advantages of edible films and coatings is their eco-friendliness. Being biodegradable, the films contribute to reducing the environmental impact associated with conventional plastic packaging, addressing concerns related to plastic waste and environmental pollution.

Biopolymers consist of repeating units of a primary organic molecule, forming chain-like structures. Their carbon-based composition makes them biodegradable, making them well-suited for developing a wide range of materials in industries like cosmetics, pharmaceuticals, textiles, paper, and food. Over the last decade, there has been a notable surge in research efforts focused on biopolymers, especially those derived from recyclable sources. Among the various biopolymers, notable examples include starches, celluloses, pectin, chitosan, polylactic acids, polyhydroxy acids, gelatine, carrageenan, and more [9,10]. These biopolymers offer viable alternatives to traditional petroleum-based polymers and draw significant interest across multiple sectors. In addition to their biodegradability, these polymers can be extracted from food and animal wastes [11,12] and bacterial cultures [13] with high output, making them even more suitable for developing sustainable packaging materials.

Biopolymers exhibit remarkable versatility due to their ability to absorb significant amounts of water, their film-forming properties, high encapsulation efficiency (for drugs or preservatives), and their suitability for creating gas and moisture barrier films through inter-polymer connections. The crosslinking feature and the overall characteristics of these films can be enhanced by controlling the linkages within the polymer matrix. This can be achieved through various methods, including crosslinking the polymer with the help of crosslinking agents involving both physical and mechanical processes. Crosslinking can be initiated through chemical agents such as calcium chloride [14], citric acid (CA) [15], polyvinyl alcohol (PVA) [16], divinyl sulphone (DVS) [17], or by chemical reactions induced by factors like pressure, temperature, pH changes, irradiation, and more. These crosslinking agents play a crucial role in promoting stronger covalent linkages within the polymer matrix, enhancing the overall properties of the biopolymer films [18,19]. Furthermore, these mechanically robust crosslinked polymers can serve as effective carriers for encapsulating bioactive substances (BSs), allowing for the creation of product-specific functional packaging films with preservative properties.

The concept of biodegradable packaging materials based on polymers has garnered significant attention in the food industry, making it a trending research focus for food scientists. The distinctive feature of these materials lies in their outstanding flexibility and adaptability, enabling them to accommodate the specific requirements of various food products. This advantage enables continuous progress in the development of improved materials each year. However, when striving to create an effective packaging material, the expectation is to match the properties of synthetic polymers such as polypropylene or polystyrene. If not identical, it is entirely feasible to achieve similar desirable properties by incorporating crosslinking agents into the polymers. This strategy serves to enhance the overall characteristics of the biodegradable packaging materials, making them a more viable and competitive alternative to traditional synthetic polymers.

Carboxymethyl cellulose (CMC) is a polysaccharide derived from glucose and is characterised by its linear branching structure. This abundant natural resource is found in various forms throughout nature, serving as a renewable and sustainable material. It plays a crucial role as a structural component in plants, crops, bacterial biomass, and animal biomass [20,21,22]. In an aqueous solution, the sodium salt of carboxymethyl cellulose (CMCNa) undergoes dissociation, breaking into carboxyl anions (COO^−^) and sodium cations (Na^+^). As water interacts further with the polymer, the anions attract more water molecules, causing the solution to thicken and eventually reach a gel-like state. Simultaneously, the free sodium cations disperse throughout the medium, balancing the net negative charge of the solution. At low concentrations, CMCNa molecules maintain a rod-shaped and extended configuration. However, as the concentration increases, these molecules begin to overlap, forming coils, and eventually become entangled with one another. This behaviour explains the thickening and gelling properties of CMCNa in aqueous solutions, making it useful in various applications such as food products, pharmaceuticals, and cosmetics [23]. CA is one of the most experimented crosslinkers used for CMC due to its advantages, including non-toxicity, ready availability, and practicality [24]. When CA is introduced to a solubilised CMC, it forms citrate ions that establish covalent linkages within the polymer network, connecting with carboxyl ions. Simultaneously, these citrate ions interact with sodium cations, limiting their free movement and leading to an increase in the solution’s viscosity, resulting in a gel-like consistency. The free movement of sodium cations is further restricted as they become trapped within the covalent bonds of the polymer matrix. This dehydration process has been reported to be facilitated at several time-temperature combinations such as 80 °C-overnight or 8 h [25] 50 °C for 15 h [26], and 100 °C for 1 h [27]. The presence of CA ultimately results in a more interconnected and rigid polymer structure due to the esterification of the hydroxyl groups on the polymer chains [25,28].

CMC is a highly studied biopolymer, primarily because of its ready availability. Within the food industry, it serves various functions, such as acting as a humectant to retain moisture and a thickening agent to enhance texture and consistency. While recent research has highlighted its potential as an edible coating for food products, it is worth noting that the exploration of CMC as an edible packaging material, particularly with a focus on food-specific applications, remains relatively limited [29,30]. This presents an area of opportunity for further investigation and innovation in the realm of sustainable and edible food packaging.

In light of prevailing trends in the literature, the primary objective of this research was to create films by crosslinking CMCNa with CA. Additionally, this study incorporated four different bioactive substances (BSs) for encapsulation purposes, namely thyme essential oil (TEO), clove essential oil (CEO), oregano essential oil (OEO), and the pure component eugenol (PCE). These BSs were individually added to the films, as well as in combination (1:1 ratio), to enhance the barrier properties of the resulting films. The selection of these BSs was based on their recognised efficacy as antioxidant and antimicrobial agents, as reported in the existing literature [1,31].

## 2. Materials and Methods

### 2.1. Chemicals and Reagents Used

Citric acid monohydrate was purchased from Lachner chemicals (Neratovice, Czech Republic), anhydrous glycerol was purchased from Fluka Chemicals (Honeywell, (Charlotte, NC, USA)); 2,2-diphenyl-1-pricylhydrazyl (DPPH), CMCNa (MW = 90,000 Da, D.S = 0.7), TWEEN^®^ 80 (T80), thyme essential oil (TEO), clove EO (CEO), oregano EO (OEO), and pure component eugenol (PCE) were all purchased from Sigma Aldrich (Burlington, MA, USA). All the chemicals were of analytical grade. Distilled water (DW) was used for all experiments.

### 2.2. Experiment Design and Statistical Analysis

Using central composite design (CCD), 13 randomized experiments were achieved, and response surface methodology (RSM) was utilized to evaluate effectiveness of the selected independent factors, including CMCNa concentration (*X*_1_, 3–5 g) and CA concentration (*X*_2_, 0.1–0.7 g) on the response variables namely tensile strength (*Y*_1_, kPa) and elongation at break (*Y*_2_, %). Centre point (*X*_1_ = 4 g, *X*_2_ = 0.4 g) was repeated five times to minimize pure error. Response variables were correlated to the linear (*X_i_*), quadratic (*Xi*^2^), and interaction (*X_i_X_j_*) terms of independent factors based on second-order polynomial model [32,33].

(1) $$Y_{i}=\beta_{o}+\sum \beta_{i}X_{i}+\sum \beta_{ii}X_{i}^{2}+\beta_{ij}X_{i}X_{j}$$

This equation contains constant (*β_o_*) and coefficients of main (*β_i_*), quadratic (*β_ii_*), and interaction (*β_ij_*) terms of the studied independent factors. The suitability of the created models for the response variables was evaluated using obtained values for coefficient of determination (R^2^) and adjusted coefficient of determination (R^2^-adj). The importance of the terms of independent factors was assessed using analysis of variance (ANOVA) based on *p*-value, where a small *p* value (*p* < 0.05) was chosen as a significant effect. The analysis was carried out using Design Expert, version 13 (Stat-Ease Inc., Minneapolis, MI, USA).

To visualise the effects of CMCNa and CA concentration on the mechanical strength of the films (the two response factors), three-dimensional surface plots are provided (Figure S1—Supplementary Material). Numerical optimisation was used to achieve the exact values for the amount of CMCNa and CA to prepare films with maximum tensile strength and strain. The final formula of the films was finalised out to be (*X*_1_ = 4.55 g) and (*X*_2_ = 0.1 g). The oil concentration was finalised at 3% (*w*/*v*) and glycerol at 2.3% (*w*/*v*) of the total weight of the polymer based on the literature review. Tween 80 was added in an equal amount as that of oil.

### 2.3. Film Formation

After finalising the formula, the films were prepared using the methodology described by Shen et al. [11]. A measure of 4.55 g of CMCNa was dissolved in 100 mL DW with continuous stirring till the solution was clear and uniform. The polymer solution was allowed to hydrate overnight. The next day, 3% (*w*/*v*) oil and T80 was added to the polymer solution and mixed using a Moulinex slimforce mixer (Paris, France) for 5 min. A measure of 0.1 g CA was dissolved in 2 mL DW separately and added to the emulsion and mixed again for 5 min. The prepared emulsions were allowed to stand for 1 h to remove all bubbles. The emulsion was then poured into Petri plate (d = 8.7 cm) to a thickness of 5 ± 0.05 mm (15 mL approx.). The drying was carried out in two stages: 37 °C for 7 h to evapourate water and 80 °C for 2 h to facilitate crosslinking [25].

### 2.4. Film Thickness and Opacity

The film thickness was measured using a handheld digital vernier calliper (PROTECO, Pardubice, Czech Republic) with a sensitivity of 0.01 mm. The results were reported as the mean of at least five random locations on the film. The opacity of films was determined by measuring the film absorbance at 600 nm using an ultraviolet spectrophotometer (Cecil CE 1021 spectrophotometer, Cambridge, United Kingdom) according to the method described by Wang [34]. Films were cut into 4.5 cm × 1 cm and directly placed in the test cell. An empty cell was utilised as the reference and all tests were conducted in triplicate. The following equation calculated the film opacity:

T = Abs_600_/d (2)

where Abs_600_ is the absorbance value at 600 nm and d is the thickness of the films (mm).

### 2.5. Scanning Electronic Microscopy (SEM) and Confocal Laser Scanning Microscopy (CLSM)

SEM of the material was carried out with aid of JEOL JSM-7500F Field Emission Scanning Electron Microscope (Tokyo, Japan). SEM images were recorded for the samples coated with 20 mm of Chromium.

For visualising the oil reservoirs in the film, a Zeiss LSM780 confocal microscope (Carl Zeiss, JSC, Oberkochen, Germany) equipped with a Plan-Apochromat 63 × 1.4 Oil DIC M2 was used. Recorded images were analysed using ZEN software (Carl Zeiss, JSC, Oberkochen, Germany).

### 2.6. Moisture Content Analysis

To determine the moisture content, the films were weighed before (*W*_1_) and after drying (*W*_2_). A laboratory oven (BMT-Venticell, Brno, Czech Republic) was employed at 110 °C until constant weight was reached (dry sample weight). Three replications of each film treatment were used to calculate the moisture content and calculated using the following formula:

(3) $$\%\;\mathrm{Moisture}\;\mathrm{content}=\frac{W_{2}-W_{1}}{W_{1}}\times 100$$

### 2.7. Moisture Absorption Rate

Moisture absorption was carried out by the protocol established by Shen and Kamdem [11]. The films were cut into square pieces (2 × 2 cm) and conditioned using anhydrous calcium chloride at 0% relative humidity (RH) for 48 h. The films were weighed accurately (*W*_1_), followed by the conditioning of the films in a desiccator containing potassium sulphate-saturated solution at 23 ± 2 °C and 97% RH. The weight of samples was recorded every week (*W*_2_) for 4 weeks to obtain the percentage of moisture absorption. Following formula was used to calculate the moisture uptake value:

(4) $$\%\;\mathrm{Moisture}\;\mathrm{uptake}=\frac{W_{2}-W_{1}}{W_{1}}\times 100$$

The resulting values were fit with a first-order kinetics reaction to calculate the moisture absorption rate per hour.

### 2.8. Water Vapour Permeability (WVP) and Water Vapour Transmission Rate (WVTR)

WVP of the films was measured according to the standard method ASTM E96-95 [35]. Films were fixed on top of Payne permeability cups (Elcometer, Manchester, UK) containing (10 ± 1 g) anhydrous calcium chloride. The cups were weighed and then placed in a desiccator filled with distilled water at 25 °C and relative humidity of 75%. The weight of test cups was measured after 48 h. The water vapour permeability (*WVP*) in gm^−1^ s^−1^ Pa^−1^ and water vapour transmission rate (*WVTR*) in gm^−2^h in were calculated through Equations (5) and (6):

(5) $$WVP=\frac{∆W\times d}{A\times ∆P}$$

(6) $$WVTR=\frac{∆W}{A\times t}$$

where Δ*W* is the weight change before and after the test (g); *A* is the test area (m^2^); *d* is the thickness of films (mm); Δ*P* is the partial pressure difference across the films (kPa); *t* is the test time (h). Every test was replicated three times.

### 2.9. Fourier Transform–Infrared (FT-IR) Spectroscopy

Infrared analysis was performed on Bruker IFS 55 FT-IR (Billerica, MA, USA) spectrometer equipped with attenuated total reflection accessory—Golden Gate—with diamond as the ATR element, single-reflection, and an incident angle of 45°. Measurements were recorded in the wavenumber range of 4000 to 500 cm^−1^, with an interval of 4 cm^−1^. The observed spectra were ATR corrected by Kubelka-Munk conversions. Chemometric analyses of the ATR-FT-IR spectra were performed using Opus software (Bruker, Billerica, MA, USA). The graphs were normalised using OriginPro 9.0 (OriginLab, Northampton, MA, USA).

### 2.10. Mechanical Properties

Tensile strength (TS), elongation at break (EB) and Young’s modulus of elasticity (EM) were measured on Shimadzu AGS-100kNX (Kyoto, Japan) according to the ASTM standard method D882 [36]. The applied strain rate was of 5 mm/min and the sample size with the dimensions 20 mm × 30 mm× 0.1 mm (length × width × thickness) was used. All measurements were carried out in triplicates.

### 2.11. Thermogravimetric Analysis

Thermogravimetric analysis (TGA) was carried out on Discovery SDT 650B-TA Instruments (New Castle, DE, USA) according to the ASTM standard method D3418 [37]. The samples were cut into tiny circles to fit the aluminium pans. Three film layers were placed (weight = (10.0 ± 1.2) mg) in an open pan. The conditions of the experiment were set as follows: heat flow 10 °C/min and dynamic atmosphere of nitrogen (N_2_ = 50 mL/min); temperature range was from 30 °C to 600 °C. The data obtained were then analysed using the Thermal Analysis Universal 2000 version 4.5A software (TA Instrument, New Castle, DE, USA).

### 2.12. Antioxidant Activity

The release of the active components of the prepared films into four food simulants was carried out at the temperatures of 4 °C and 25 °C [38]. Food simulants were selected according to the European regulations [39]: 3% *v*/*v* acetic acid (AA-Food stimulant B) as an acidic food simulant (pH 4.5); 10% *v*/*v* ethanol (10% EtOH-Food stimulant A) as an aqueous food simulant; and 50% *v*/*v* ethanol (50% EtOH-Food stimulant D1) as a simulant for foods with a lipophilic character. Distilled water (DW) was also selected as a control food simulant for comparison. To carry out the release of active components, 4 × 4 cm^2^ film specimens were immersed in 10 mL of simulant solution for 2 h at predefined temperatures. The liquid was then separated from the semi-dissolved film/film residues and centrifuged (DM0412 Clinical centrifuge, (DLab, Lonay, Switzerland)) to separate any solid components. The antioxidant activity was measured with a standard antioxidant assay using DPPH at 517 nm wavelength. The percentage inhibition values were calculated according to the following equation:

(7) $$\%\;Inhibition=\frac{A_{blank}-A_{sample}}{A_{blank}}\times 100$$

where *A_blank_* is the absorbance of the methanolic solution of DPPH and *A_sample_* is the absorbance of the samples.

## 3. Results

### 3.1. Film Appearance, Thickness, and Optical Properties

The sample codes and the results for film thickness and transparency are summarized in Table 1, while the data for light transmittance are presented in Table 2. The thickness of the films ranged from 0.08 to 0.1 mm, and there were no significant differences observed (*p* < 0.05). In the context of food quality assessment, visual inspection is a critical aspect, and packaging materials with good transparency properties are highly desirable. It is worth noting that higher transparency values typically correspond to lower UV-barrier properties [40]. Visual inspection of the films revealed that these were suitably clear and smooth, without any cracks or surface irregularities (Figure S2). Interestingly, all film samples containing oil exhibited better UV-barrier properties when compared to the control sample. Furthermore, samples containing two different oils displayed an even stronger UV-barrier effect compared to films with only one type of oil. Specifically, the TE sample exhibited the most effective UV barrier properties, featuring the lowest transparency value and the highest level of opacity. The opacity observed in films containing oils has been attributed to the presence of a miscible phase that contributes to the opaqueness of the films as they dry, as noted by Dashipour et al. [41]. According to Cai [42], the opacity of films containing oils is a result of preceding light scattering due to the even distribution of the oil droplets within the film matrix.

### 3.2. SEM and CLSM

**Figure 1:**
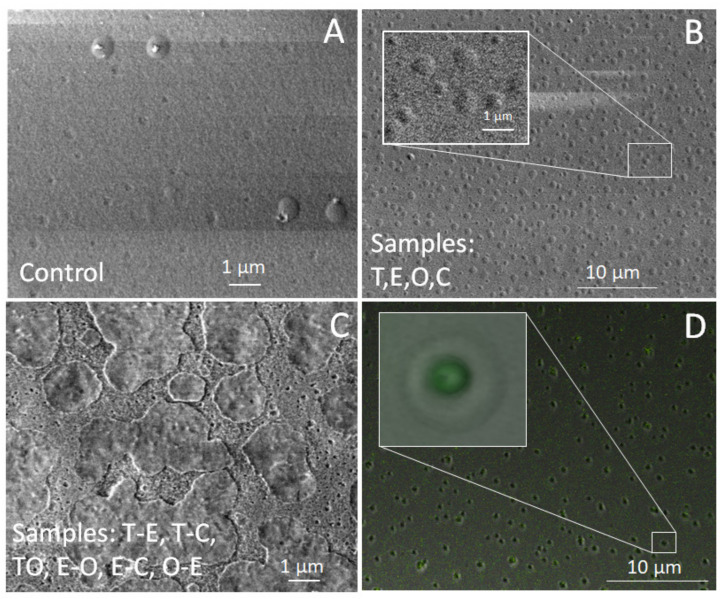
SEM (**A**–**C**) and CLSM images (**D**) of the prepared films.

The surface characteristics of the prepared films, as examined using SEM (scanning electron microscopy) and CLSM (confocal laser scanning microscopy), are depicted in Figure 1. The control sample exhibited a relatively smooth surface (Figure 1A). In contrast, all the other samples displayed a rough surface with crater-like deposit structures (Figure 1B), resulting from the encapsulated oil micro-reservoirs within the polymer films. As an addition, as shown in Figure 1B, the latter reservoirs, when analysed using SEM in a vacuum, created typical nucleation and growth patterns that are very well known from polymer physics [43]. Interestingly, for the films prepared with two BSs, we observed interpenetration-like phase patterns of distribution matter, reflecting the spinodal-like polymer decomposition patterns (Figure 1C) [43,44].

Using CLSM imaging (Figure 1D), we confirmed the above-mentioned uniform planer distribution of oil within the polymer matrix, mirroring the visualisation previously observed by SEM. Moreover, the structure of the encapsulated oil was observed to have a thick outer coating. These observations were in excellent alignment with the findings of Hamal et al. [45]. They reported that oil encapsulation within cellulose resulted in the oil being surrounded by a porous cellulose hydrogel shell [45].

### 3.3. Moisture Content and Moisture Absorption Rate

**Figure 2:**
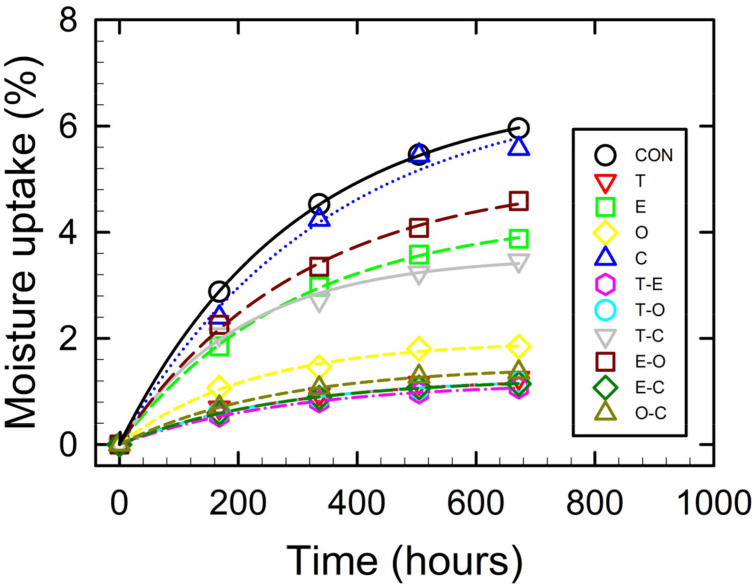
Moisture absorption rate of the prepared film samples.

The results of the moisture content analysis performed at RH = 97% are presented in Table 1, while the absorption rates are illustrated in Figure 2. The moisture content of the films ranged from 0.04% to 0.06% by weight, with no significant differences observed (*p* < 0.05). These values were notably lower compared to those reported for films prepared with pure CMCNa [41]. This variation can be attributed to the presence of the crosslinking agent (CA) in the films.

It is assumed that CA interacts with the carboxylic acid groups on the polymer chains, catalysed by heat, resulting in the removal of water molecules through the dehydration reaction. The moisture absorption content depends on several factors, including the initial moisture content of the films, the degree of crosslinking, the interactions between the film’s ingredients and moisture, the concentration of polymer in the films, the strength of the polymer chains, and the availability of glycerol molecules to facilitate moisture diffusion.

In the literature, significantly higher moisture absorption values have been reported. For example, Ghanbarzadeh et al. [46] reported a value of 34%, Shen et al. [11] reported 55.71%, and Bazzar et al. [47] reported a 19% moisture absorption value for the pure CMCNa control sample. In contrast, the control sample in the present study showed a value of 5.9% after four weeks of storage. The notable difference can be attributed to the higher concentration of CMCNa used in this study, resulting in denser films due to the successful crosslinking of the polymer matrix. The reference studies used a lower concentration of 1% CMCNa without the presence of the crosslinking agent, leading to the reported higher moisture absorption values.

Among the samples under study, the control sample exhibited the highest moisture absorption rate. The other samples displayed a relatively non-uniform trend in the following order: C < EO < E < T < C/EO/T < TE. Here, sample C had the highest moisture absorption rate and TE had the lowest one. The observed high moisture absorption rates of samples C, EO, E, and TC can be attributed to the sparingly soluble nature of PCE, CEO, and TEO (with TEO being the least soluble among them). In contrast, samples O, T, OC, TO, and TE exhibited the lowest moisture absorption rates. It can be inferred that the polarity and degree of solubility of essential oils in the dispersed medium directly impact the moisture absorption kinetics of the films.

The elucidation of the observed behaviour becomes apparent when considering mixing as a consequence of the mutual competition between fundamental components of osmotic pressure. This competition arises due to the translational motion of polymers and the forces acting between monomeric units. Osmotic pressure, a consistent factor supporting mutual mixing, is contingent upon polymer density (c_p_). Concurrently, the portion of pressure originating from monomer–monomer interactions may exhibit positivity or negativity, depending on monomer density (c_m_). Given the relationship c_p_/c_m_ = 1/N, (where N is the polymerisation degree) the contribution associated with osmotic pressure remains exceedingly small relative to the influence of monomer–monomer forces. In cases where the mixing enthalpy exceeds the entropic term in significance, the phenomenon of mixing or dissolution is precluded, denoted as the mutual intolerance of polymers. Exceptional instances involve two polymers with negative mixing enthalpies, allowing for mutual miscibility without constraint. Typically, the mixing of two polymers results in a heterogeneous or micro-heterogeneous mixture, characterized by hindered separation due to the high viscosity of the mixture.

### 3.4. WVP and WVTR

**Figure 3:**
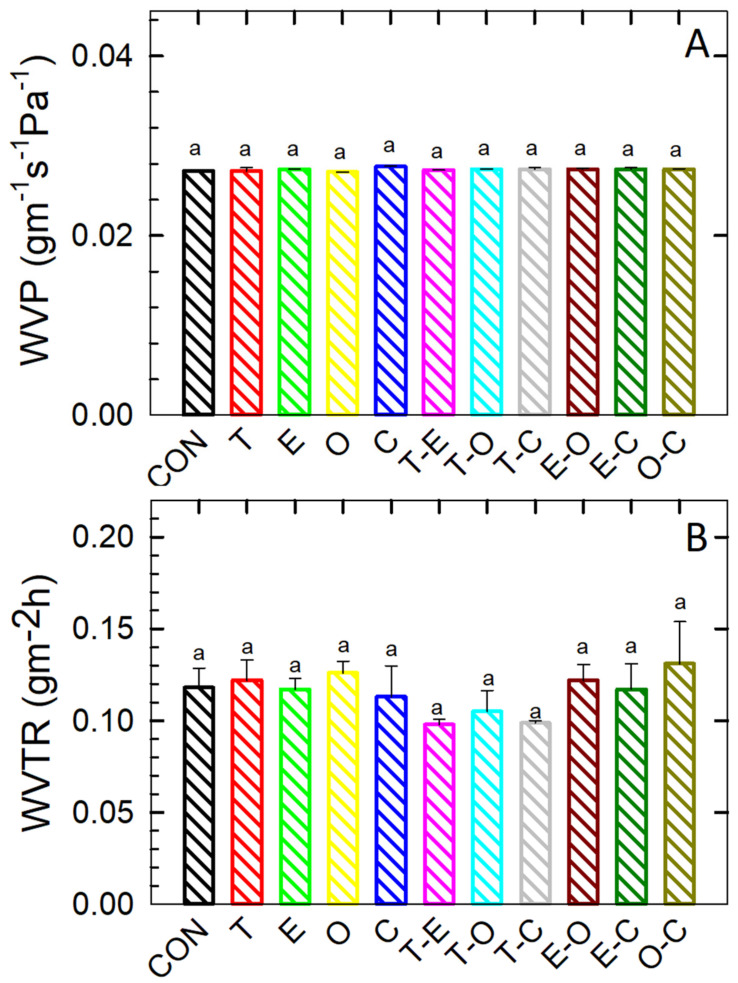
WVP (**A**) and WVTR (**B**) of the prepared film samples. Differences in the mean values among the statistical groups were tested at a significance level of *p* < 0.05. The Tukey test was applied for multiple comparisons of the mean values to assess statistical significance, i.e., to evaluate if the differences were greater than what would be expected by chance; different letters were used to indicate statistically significant differences between the values determined. The results were expressed as arithmetic mean ± standard deviation.

The water vVapour permeability (WVP) and water vVapour transmission rate (WVTR) of the films under investigation are depicted in Figure 3. Similar factors, as discussed in Section 3.3, influenced WVP. It was determined that permeability was not statistically significantly impacted by the presence or specific type of bioactive substances (*p* < 0.05). This lack of impact can be ascribed to the uniform composition of the polymer base, thereby indicating that the transport properties are controlled by a diffusion process.

The WVP values displayed a consistent similarity across all samples, in stark contrast to the results observed in moisture absorption studies. This disparity is attributed to fluctuations in humidity conditions during the analysis, conducted at RH = 75%. Another factor to consider is the uniform distribution of the non-polar and dispersive nature of bioactive substances (BSs), which establishes a barrier, repelling polar water vapours from the interface. Nevertheless, this phenomenon was not evident in WVTR experiments. It is hypothesized that adhered water vapours underwent a condensation phase transition, followed by the gradual swelling of the base polymer layer.

The acquired diminished values of WVP and WVTR, in comparison to published data [13,29,46,47,48], signify the commendable barrier properties exhibited by the CMC films. As elucidated earlier, the enhancement in barrier properties is presumably attributable to the formation of chemical bonds within the system facilitated by the inclusion of a crosslinking agent.

### 3.5. FT-IR

**Figure 4:**
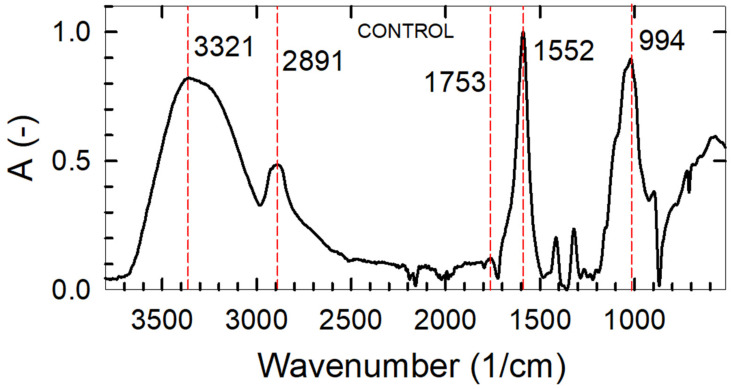
FTIR analysis of the control sample with major peaks.

The results of FT-IR analyses are depicted in Figure 4. The major peaks for all the samples were in the wavenumber range of 3300–2800 cm^−1^ (−OH stretching for CMCNa) and 1100–1500 cm^−1^ (C-O stretching) (Figure 4 and Figure S2 (Supplementary Material)). The distinct intense peak at 1552 cm^−1^ wavenumber was attributed to C=O bond stretching vibrations of cellulose, as reported in the studies of Ghorpade et al. and Ayouch et al. [15,28]. The low-intensity peak at 1753 cm^−1^ wavenumber was assigned to the ester bonds present between CA and hydroxyl groups of the cellulose derivative [23]. Additionally, the peak at 1753 cm^−1^ wavenumber should be ascribed to the carboxyl group stretching mode from glycerol [49]. Minor peaks observed at 994 cm^−1^ (OH bending) and 2891 cm^−1^ wavenumbers were assigned to C=O groups of glycerol as well [49].

For the films with bioactive components, the difference spectra against the control specimen were calculated. The major constituents of the bioactive components used were thymol, carvacrol, p-cymene, eugenol, and its derivatives. All the samples were found to have the hydrogen-bonded OH bonds and their representative stretching mode vibrations bands in the wavenumbers range of 3330 to 3360 cm^−1^ [50]. The only exception to this finding was recorded for the sample TC. Observed bands at 863 cm^−1^ wavenumbers were assigned to thymol [51]. This result was consistent with all the samples containing TEO. The absorption peak at 1405 cm^−1^ was attributed to p-cymene [52], which was clearly distinguishable in samples T and TE. However, it was not present in the samples TO and TC. The intense peak observed at 1980 to 2160 cm^−1^, distinct for all samples containing TEO, might be also related to the shifting of the corresponding thymol IR absorption band. Other peaks reported for thymol were observed at 1585 cm^−1^, 863 cm^−1^, 945 cm^−1^, 1087 cm^−1^, and 1289 cm^−1^ wavenumbers [53]. Shifting of bands was noted also for carvacrol in samples containing oregano EO. Carvacrol shows clear distinct peaks at 1437 cm^−1^ [54] in pure Oregano EO (Figure S3-Supplementary File). It was also present between 1950–2100 cm^−1^ wavenumbers in all the samples containing Oregano EO. The major component of clove EO is eugenol and its derivatives. The distinctive bands of pure component eugenol were present at 1506 cm^−1^ wavenumber indicative of C=C aromatic carbon double bonds moieties [55]. However, this region was found to be shifted to 2183 cm^−1^, 2167 cm^−1^, 2010 cm^−1^, 2151 cm^−1^, and 2161–2191 cm^−1^ wavenumbers for the samples of C, TE, TC, EO, and EC, respectively. In the sample EC, a major negative peak at 1560 cm^−1^ was also observed. This zone corresponds to the carboxyl group in CMCNa [56].

### 3.6. Mechanical Properties

**Figure 5:**
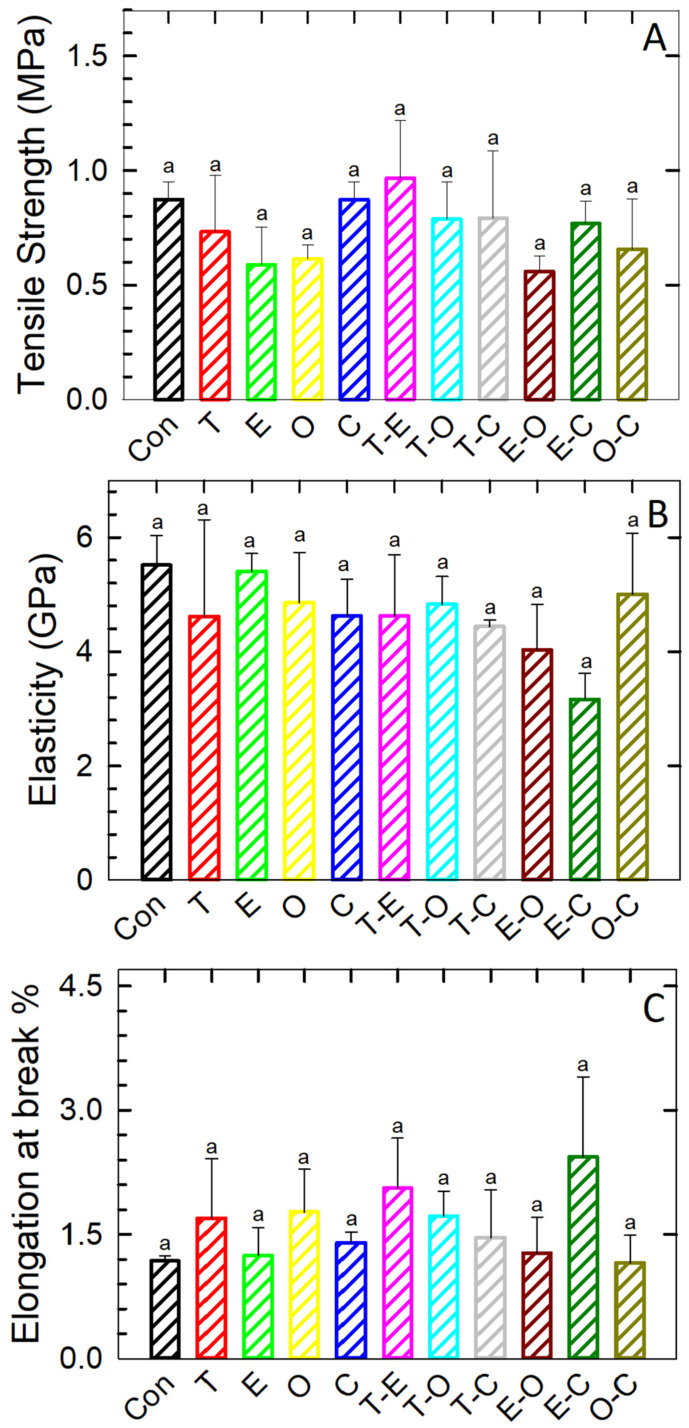
Tensile strength (**A**), Young’s modulus of elasticity (**B**), and elongation at break (**C**) of the film samples. Differences in the mean values among the statistical groups were tested at a significance level of *p* < 0.05. The Tukey test was applied for multiple comparisons of the mean values to assess statistical significance, i.e., to evaluate if the differences were greater than what would be expected by chance; different superscript letters were used to indicate statistically significant differences between the values determined. The results were expressed as arithmetic mean ± standard deviation.

The results for tensile strength (TS), elongation at break (EB), and Young’s modulus of elasticity (*E*) are presented in Figure 5. The TS of film samples containing bioactive substances (BSs) was either lower or comparable to that of the control sample, with no statistically significant difference observed (*p* < 0.05). These outcomes align with those of previous studies [41,46,57], which have consistently reported a reduction in TS with the addition of essential oils (EOs). Notably, Simsek et al. [48] reported an increase in TS, attributing it to the crosslinking effect of essential oils with the CMC polymer. In accordance with the literature and our assumptions, the observed reduction in TS with EOs is attributed to the disruption of polymer–polymer linkages. This disruption is caused by the inclusion of encapsulated oil nano reservoirs within the polymer films’ matrix, leading to a weakening of mutual entanglements and cross-linking.

Simultaneously, the low TS is also attributed to the crosslinking effect of citric acid (CA). Citrate ions (C_6_H_5_O_7_^3−^) form rigid covalent bonds with COO^−^ and Na^+^ ions, limiting their free mobility. This inhibition disrupts intermolecular hydrogen bonds, ultimately reducing the film’s elasticity and increasing its plasticity, as evidenced by decreased EM compared to the control samples. This effect is further supported by increased EB, as depicted in Figure 5B,C. EM measures the toughness of the film under pressure and indicates its extent of deformation, with the highest values observed for the control sample. In contrast, samples containing BSs exhibited similar or lower *E* values, with no statistically significant difference (*p* < 0.05) among them. A higher *E* value correlates with lower EB, consistent with the findings of this study.

### 3.7. Thermogravimetric Analysis (TG)

**Figure 6:**
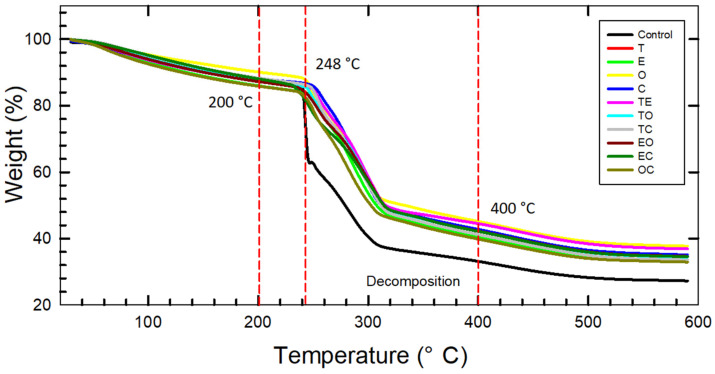
Thermogravimetric analysis of the film samples presenting major events.

The results of TG are presented in Figure 6, and they reveal three significant temperature events related to weight loss in the samples. The first event, occurring in the temperature range of 30–200 °C, was attributed to the loss of moisture and volatile compounds from the samples, resulting in a total weight loss of 9–13% for the samples. These observations in the first event align with the values in the literature [28]. The second major event took place in the range of 200–400 °C, resulting in a weight loss of 43–54%. Similar weight loss values were reported by Sotolářová [58] for CMC films crosslinked with CA, and it was claimed that crosslinking improves the thermal stability of the films. Notably, a rapid reduction in weight for control samples at 248 °C was associated with the loss of COO^−^ moieties from the CMCNa [15,59], followed by the peak at 309 °C ascribed to the cellulose depolymerization [28]. The final event, occurring in the range of 400–600 °C, corresponded to the decomposition of the samples. There was a clear distinction observed between the control sample and the bioactive samples, with the control sample exhibiting faster decomposition. This difference can be attributed to the higher stability of films resulting from the crosslinking effect of the BSs.

### 3.8. Antioxidant Activity

**Figure 7:**
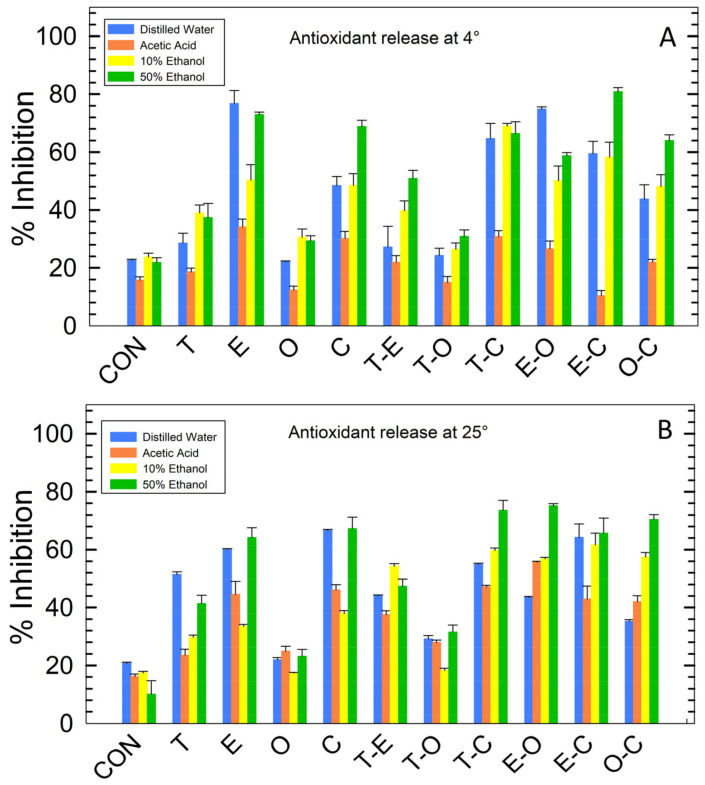
Antioxidant activity presented as % inhibition at 4 °C (**A**) and 25 °C (**B**) for the film samples.

The results of antioxidant activity, as measured at 4 °C and 25 °C, are depicted in Figure 7, while detailed tabular data are provided in Tables S1 and S2 (refer to the Supplementary Data). Considerable variation in the activity of the films was observed at both temperatures. The highest activity at 25 °C was exhibited by sample EO (in AA and 50% EtOH) and sample EC (in DW and 10% EtOH). Conversely, sample O demonstrated activity comparable to the control sample, implying poor or zero solubility in AA media. In DW, 10% EtOH, and 50% EtOH, sample O exhibited the least activity, while sample T displayed the lowest activity in AA media. At 4 °C, the films with the highest activity were sample E (in DW), EC (in 50% EtOH), and TC (in AA and 10% EtOH). Conversely, sample T (in AA), TO (in 10% EtOH), and sample O (in DW and 50% EtOH) demonstrated the lowest activity among all the bioactive film samples. The antioxidant efficacy of the films was hindered by the varying solubility of the films and the BCs in the respective media and the temperature of the media. These results, which directly indicate the suitability of the films, underscore their efficiency at different temperatures and in various media.

## 4. Conclusions

In this study, the suitability of CMC chemical crosslinking by CA as a tool for the preparation of packaging films was demonstrated. Furthermore, the embedding of bioactive substances in the packaging film was substantiated as a means to enhance the overall quality of the food product. Enhanced light opacity, particularly in sample TE with the highest value, was observed in comparison to films containing only one bioactive substance. Morphological analysis confirmed the uniform distribution of embedded bioactive substances within the films. Analysis of moisture absorption rates revealed significantly lower values for films with bioactive substances compared to the controlled sample, suggesting their applicability in the food packaging industry for moisture-laden foods. While WVP and WVTR analyses indicated no significant impact of the crosslinking agent, the obtained values surpassed those reported in the literature, attributed to the higher CMCNa content. These results underscore the films’ suitability for high-moisture conditions. Mechanical properties analysis aimed at establishing suitability for specific food applications demonstrated that sample TE exhibited the highest mechanical strength, while sample EC displayed the highest elongation at break, consistent with the lowest Young’s modulus of elasticity. This study establishes the potential use of the prepared films for packaging fresh meat and related products. However, there remains a scope for improvement in mechanical properties, warranting further research in this domain.

## Figures and Tables

**Table 1:** Sample codes, appearance, film thickness, film opacity and moisture content of the studied specimens.

Sample	Code	Thickness (mm)	Transparency (-)	Moisture Content (%)
Control	Con	0.08 ± 0.001 ^a^	0.60 ± 0.14 ^a^	0.0821 ± 0.018 ^a^
Thyme	T	0.10 ± 0.001 ^a^	0.96 ± 0.12 ^a^	0.0614 ± 0.018 ^a^
Eugenol	E	0.08 ± 0.002 ^a^	0.84 ± 0.06 ^a^	0.0649 ± 0.019 ^a^
Oregano	O	0.08 ± 0.001 ^a^	1.03 ± 0.15 ^b^	0.0749 ± 0.016 ^a^
Clove	C	0.08 ± 0.001 ^a^	0.92 ± 0.03 ^b^	0.0612 ± 0.019 ^a^
Thyme-Eugenol	T-E	0.10 ± 0.002 ^a^	1.38 ± 0.22 ^bc^	0.0741 ± 0.017 ^a^
Thyme-Oregano	T-O	0.08 ± 0.001 ^a^	0.98 ± 0.09 ^bc^	0.0867 ± 0.019 ^a^
Thyme-Clove	T-C	0.09 ± 0.001 ^a^	1.24 ± 0.09 ^bc^	0.0704 ± 0.014 ^a^
Eugenol-Oregano	E-O	0.09 ± 0.001 ^a^	1.26 ± 0.26 ^bc^	0.0679 ± 0.012 ^a^
Eugenol-Clove	E-C	0.08 ± 0.001 ^a^	0.85 ± 0.03 ^d^	0.0732 ± 0.015 ^a^
Oregano-Clove	O-C	0.09 ± 0.001 ^a^	1.07 ± 0.14 ^d^	0.0634 ± 0.016 ^a^

Results are given as arithmetic mean ± standard deviation of three replicates. The values followed by the same letters in the same raw are not significantly different at significance level of *p* ≤ 0.05 by Tukey test.

**Table 2:** Light transmittance values at various wavelengths (nm) of the studied samples.

Sample	Light Transmittance %							
	200 nm	280 nm	350 nm	400 nm	500 nm	600 nm	700 nm	800 nm
Con	0	36.18 ± 2.26 ^a^	71.69 ± 2.09 ^a^	81.92 ± 1.59 ^a^	87.65 ± 2.54 ^a^	87.06 ± 2.92 ^a^	87.77 ± 1.38 ^a^	88.20 ± 2.86 ^a^
T	0	13.52 ± 5.45 ^b^	62.20 ± 5.55 ^a^	70.64 ± 5.06 ^a^	73.22 ± 0.38 ^b^	80.12 ± 2.21 ^a^	81.10 ± 1.31 ^b^	83.65 ± 2.86 ^a^
E	0	5.49 ± 0.81 ^b^	60.45 ± 1.43 ^a^	74.53 ± 1.11 ^a^	79.80 ± 1.02 ^b^	82.29 ± 1.21 ^a^	83.30 ± 0.40 ^b^	84.79 ± 1.17 ^a^
O	0	15.85 ± 8.556 ^b^	59.24 ± 3.95 ^a^	67.52 ± 2.68 ^a^	73.87 ± 2.46 ^b^	78.79 ± 2.75 ^b^	79.90 ± 3.05 ^b^	83.12 ± 1.48 ^b^
C	0	15.99 ± 3.24 ^b^	62.41 ± 3.95 ^a^	71.21 ± 2.83 ^a^	77.51 ± 0.98 ^c^	80.91 ± 0.64 ^b^	82.60 ± 1.16 ^ab^	85.83 ± 0.30 ^ab^
T-E	0	2.90 ± 2.54 ^bc^	46.75 ± 8.11 ^b^	58.38 ± 7.75 ^b^	68.79 ± 5.39 ^d^	72.78 ± 3.85 ^c^	76.92 ± 3.90 ^b^	81.19 ± 1.45 ^b^
T-O	0	11.50 ± 1.48 ^bc^	60.55 ± 1.69 ^c^	69.94 ± 1.45 ^ab^	75.11 ± 1.28 ^d^	79.81 ± 1.78 ^c^	82.23 ± 1.14 ^b^	84.07 ± 0.29 ^ab^
T-C	0	4.20 ± 1.85 ^bc^	52.85 ± 2.75 ^c^	64.98 ± 1.87 ^ab^	70.37 ± 1.65 ^d^	75.05 ± 1.56 ^c^	78.41 ± 1.35 ^b^	81.28 ± 0.18 ^b^
E-O	0	3.08 ± 2.49 ^bcd^	53.26 ± 6.50 ^c^	63.76 ± 7.33 ^ab^	69.77 ± 4.88 ^d^	74.90 ± 4.55 ^c^	77.98 ± 3.77 ^b^	82.14 ± 2.01 ^b^
E-C	0	5.50 ± 2.60 ^bcd^	56.69 ± 2.02 ^c^	72.84 ± 1.8 ^ab^	79.25 ± 0.96 ^e^	82.22 ± 0.56 ^d^	83.17 ± 0.66 ^ab^	85.31 ± 0.70 ^ab^
O-C	0	6.44 ± 4.70 ^bcd^	55.87 ± 3.55 ^c^	68.20 ± 2.10 ^ab^	75.31 ± 2.76 ^e^	78.19 ± 2.67 ^d^	80.87 ± 2.52 ^b^	82.41 ± 0.87 ^b^

Results are given as arithmetic mean ± standard deviation of three replicates. The values followed by the same letters in the same raw are not significantly different at significance level of *p* ≤ 0.05 by Tukey test.

## Data Availability

Data will be available upon reasonable request.

## Supplementary Materials

The following supporting information can be downloaded at: https://www.mdpi.com/article/10.3390/foods12244454/s1, Figure S1: Response surface plots representing the effect of CMCNa and CA concentration on tensile strength (A) and elongation at break (B); Figure S2: Physical appearance of the film samples; Figure S3: FT-IR analysis of pure bioactive substances; Figure S4: FT-IR analysis of films with bioactive components; Table S1: Mechanical properties of the film samples; Table S2: Antioxidant activity presented as % inhibition at 4 °C; Table S3: Antioxidant activity presented as % inhibition at 25 °C.
