# Supplementary material for: Physicochemical Characterisation of Polysaccharide Films with Embedded Bioactive Substances
Source: Foods. 2023 Dec 12;12(24):4454. doi: 10.3390/foods12244454 (PMC10743232; doi:10.3390/foods12244454)
Supplement: Supplementary file 1 [file foods-12-04454-s001.zip › foods-2768526-supplementary.pdf]

foods

Supplementary Material

Physicochemical Characterisation of Polysaccharide Films with Embedded Bioactive Substances

Shweta Gautam[1], Lubomir Lapcik[1,2,*], Barbora Lapcikova[1,2], David Repka[2], Lilianna Szyk-Warszyńska[3]

[1] Department of Foodstuff Technology, Faculty of Technology, Tomas Bata University in Zlín, Nam. T.G. Masaryka 5555, 76001 Zlín, Czechia; gautam@utb.cz (S.G.); lapcikl@seznam.cz (L.L.); lapcikova@utb.cz or barbora.lapcikova@upol.cz (B.L.)
[2] Department of Physical Chemistry, Faculty of Science, Palacky University in Olomouc, 17. Listopadu 12, 771 46 Olomouc, Czechia; david.repka@upol.cz (D.R.)
[3] Jerzy Haber Institute of Catalysis and Surface Chemistry, Polish Academy of Sciences, Niezapominajek 8, 30239 Kraków, Poland liliana.szyk-warszynska@ikifp.edu.pl (L.S.)
*Correspondence: lapcik@utb.cz or lapcikl@seznam.cz or lubomir.lapcik@upol.cz; Tel: +420-576-035-115

Citation: Gautam, S.; Lapcik, L.; Lapcikova, B.; Repka, D.; Szyk-Warszyńska, L. Physicochemical Characterisation of Polysaccharide Films with Embedded Bioactive Substances. *Foods* **2023**, *12*, 4454. https://doi.org/10.3390/foods12244454

Academic Editor: Cristóbal Noé Aguilar González

Received: 27 November 2023
Revised: 8 December 2023
Accepted: 11 December 2023
Published: 12 December 2023

Copyright: © 2023 by the authors. Licensee MDPI, Basel, Switzerland. This article is an open access article distributed under the terms and conditions of the Creative Commons Attribution (CC BY) license (https://creativecommons.org/licenses/by/4.0/).

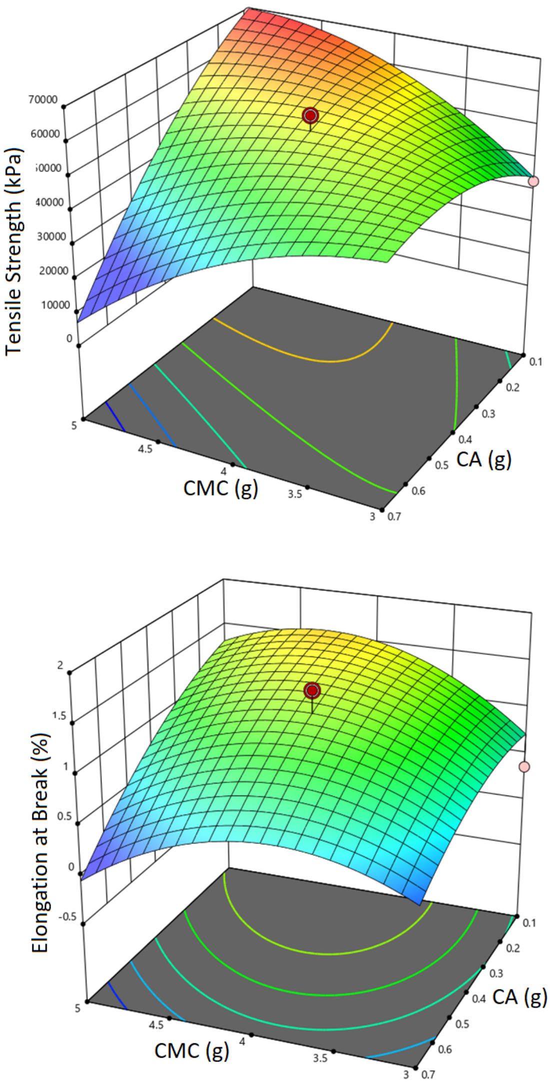

Figure S1. Response surface plots representing the effect of Sodium Carboxymethyl cellulose (CMCNa) and citric acid (CA) concentration on Tensile strength (A) and Elongation at break (B).

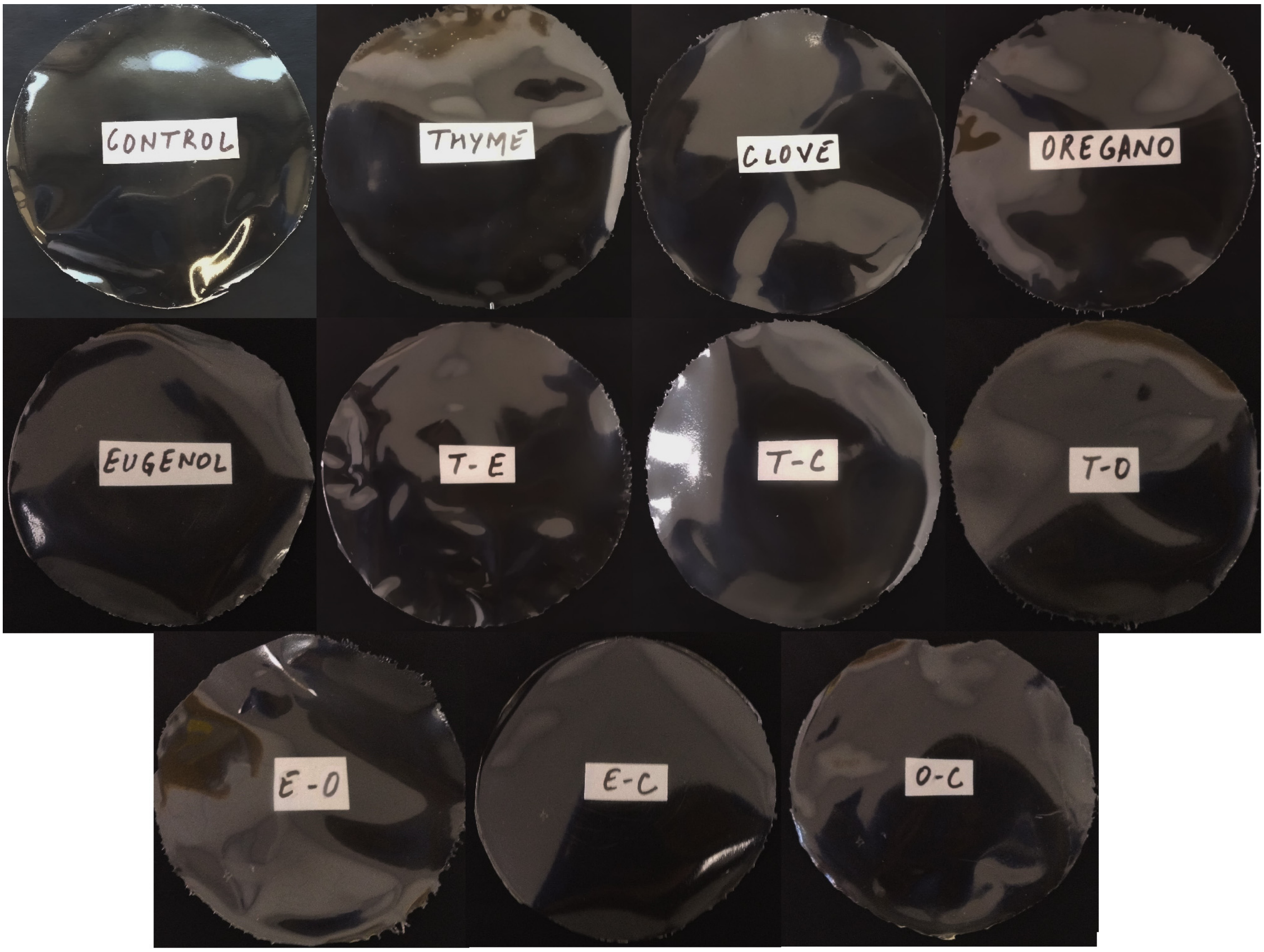

Figure S2. Physical appearance of the prepared films

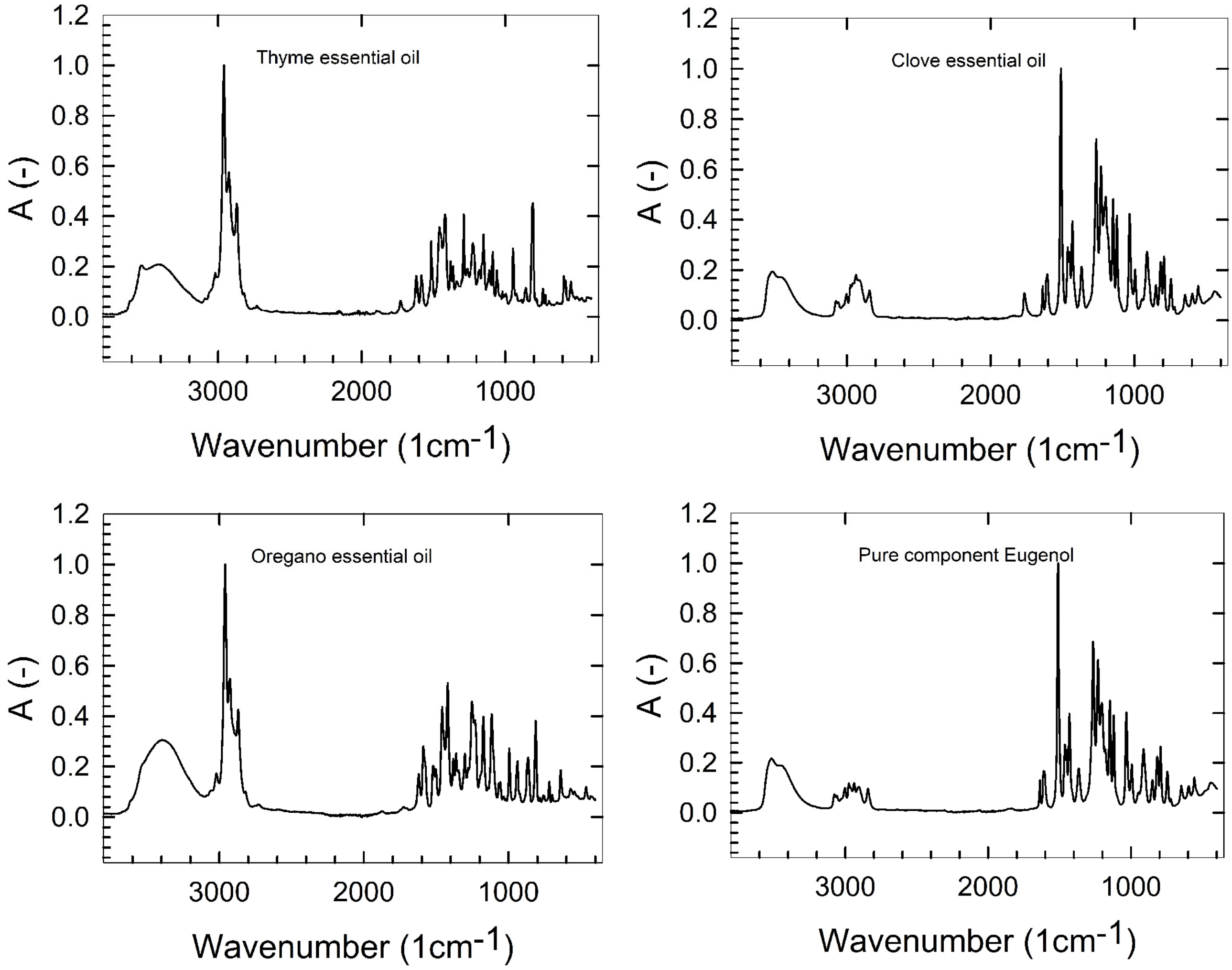

Figure S3. FT-IR analysis of the pure bioactive substances.

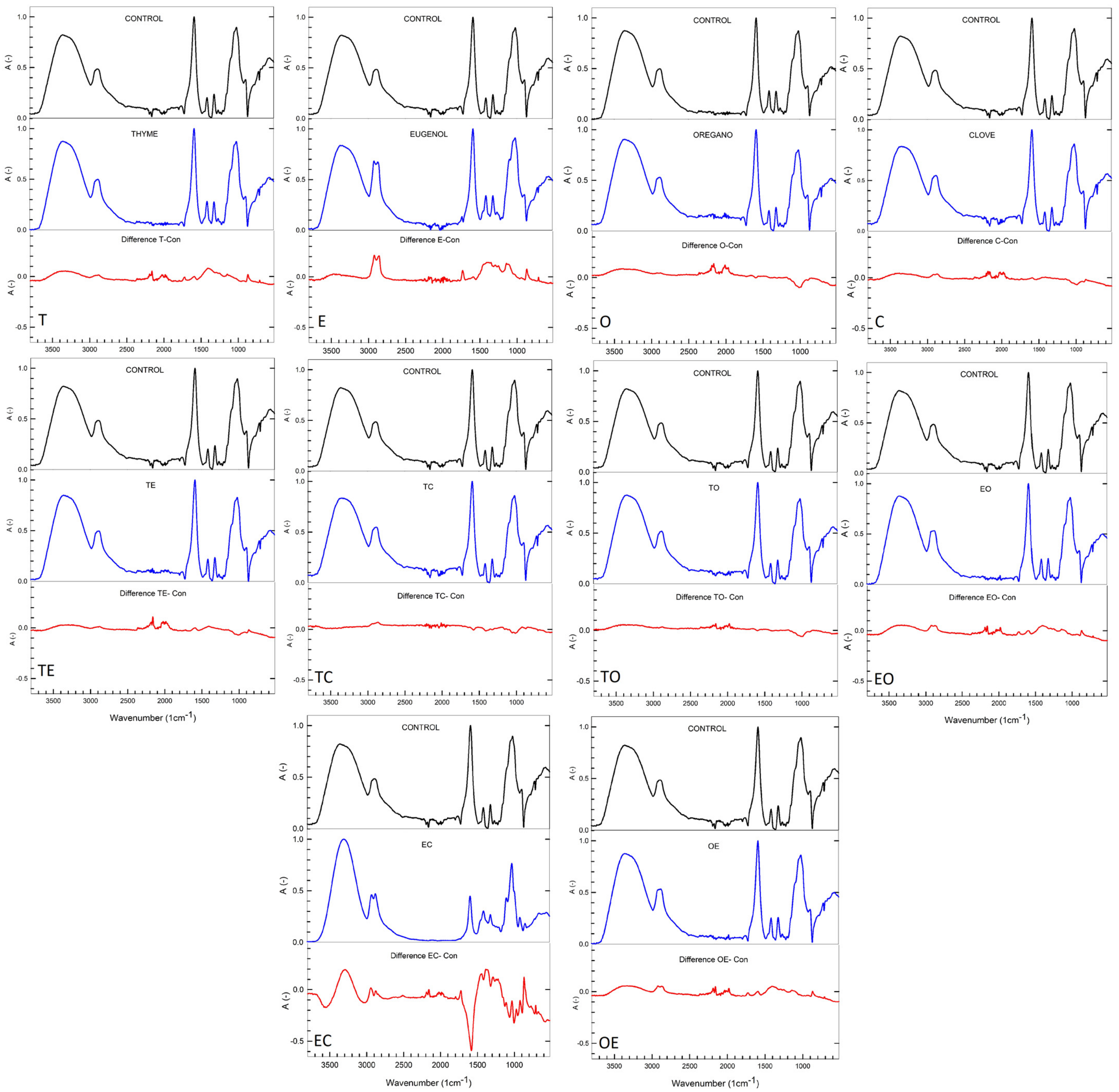

Figure S4. FT-IR analysis of films with bioactive components. The graphs are presented after subtracting the sample values from the control values to show the presence of bioactive components.

Table S1. Mechanical properties of the samples.

Sample	Tensile strength (MPa)	Elongation at break (%)	Elastic modulus (GPa)
Con	0.87 ± 0.07[a]	1.18 ± 0.06 [a]	5.51 ± 0.51 [a]
T	0.73 ± 0.24 [a]	1.69 ± 0.71 [a]	4.61 ± 1.69 [a]
E	0.58 ± 0.16 [a]	1.24 ± 0.33 [a]	5.40 ± 0.30 [a]
O	0.61 ± 0.05 [a]	1.77 ± 0.51 [a]	4.85 ± 0.87 [a]
C	0.87 ± 0.07 [a]	1.39 ± 0.12 [a]	4.62 ± 0.64 [a]
T-E	0.96 ± 0.25 [a]	2.06 ± 0.60 [a]	4.62 ± 1.07 [a]
T-O	0.78 ± 0.16 [a]	1.72 ± 0.30 [a]	4.83 ± 0.48 [a]
T-C	0.79 ± 0.29 [a]	1.46 ± 0.57 [a]	4.44 ± 0.11 [a]
E-O	0.55 ± 0.06 [a]	1.27 ± 0.43 [a]	4.03 ± 0.79 [a]
E-C	0.76 ± 0.09 [a]	2.44 ± 0.96 [a]	3.16 ± 0.45 [a]
O-C	0.65 ± 0.22 [a]	1.15 ± 0.33 [a]	5.01 ± 1.06 [a]

Results are mentioned as arithmetic mean ± standard deviation of three replicates. The values followed by the same letters in the same raw are not significantly different at the significance level of $p \leq 0.05$ by the Tukey test.

Table S2. Antioxidant activity (presented as % inhibition) of the samples at 4 °C.

Sample	DW @ 4°	AA	EtOH 10%	EtOH 50%
Con	22.62 ± 0.62[a]	15.80 ± 2.01[a]	23.77 ± 2.47[a]	21.84 ± 2.92[a]
T	28.59 ± 5.81[a]	18.55 ± 2.37[a]	38.99 ± 4.67[a]	37.38 ± 8.39[a]
E	76.77 ± 7.78[b]	34.14 ± 4.75[b]	50.33 ± 9.20[b]	72.99 ± 1.40[a]
O	22.15 ±0.45[a]	12.37 ± 2.29[a]	30.51 ± 4.99[b]	29.38 ± 2.89[a]
C	48.43 ± 5.44[c]	30.11 ± 4.27[a]	48.47 ± 10.08[b]	68.78 ± 3.85[a]
T-E	27.19 ± 12.43[a]	21.89 ± 4.04[ab]	39.77 ± 8.14[ab]	50.92 ± 3.81[a]
T-O	24.29 ± 4.37[a]	15.02 ± 3.54[ab]	26.40 ± 3.89[ab]	30.80 ± 5.11[a]
T-C	64.69 ± 9.00[b]	30.75 ± 3.59[ab]	68.97 ± 1.27[c]	66.40 ± 7.00[a]
E-O	74.84 ± 1.40[b]	26.58 ± 4.77[ab]	50.14 ± 12.31[c]	58.74 ± 1.85[a]
E-C	59.49 ± 7.35[b]	10.35 ± 3.16[b]	58.22 ± 9.08[c]	80.84 ± 2.42[b]
O-C	43.74 ± 9.94[ac]	21.94 ± 1.69[ab]	48.13 ± 10.9[ab]	64.02 ± 4.23[a]

Results are mentioned as arithmetic mean ± standard deviation of three replicates. The values followed by the same letters in the same raw are not significantly different at the significance level of $p \leq 0.05$ by the Tukey test.

Table S3. Antioxidant activity (presented as % inhibition) of the samples at 25 °C.

Sample	DW @ 25°	AA	EtOH 10%	EtOH 50%
Con	20.77 ± 0.65[a]	16.15 ± 1.47[a]	17.38 ± 0.97[a]	10.10 ± 7.96[a]
T	51.39 ± 1.65[a]	23.52 ± 3.68[a]	29.72 ± 1.26[a]	41.34 ± 5.78[a]
E	60.16 ± 0.46[b]	44.54 ± 11.2[a]	33.52 ± 1.12[a]	64.16 ± 6.80[a]
O	21.95 ± 1.40[b]	24.94 ± 2.86[a]	17.32 ± 0.37[a]	23.18 ± 4.84[a]
C	66.60 ± 0.64[c]	46.10 ± 3.12[a]	37.93 ± 1.68[a]	67.26 ± 7.92[a]

T-E	44.12 ± 0.40[b]	37.56 ± 2.17[a]	54.26 ± 1.58[a]	47.42 ± 4.12[a]
T-O	29.13 ± 2.06[b]	27.89 ± 1.42[a]	18.28 ± 1.28[a]	31.52 ± 4.85[a]
T-C	54.94 ± 0.65[b]	47.19 ± 0.94[a]	59.76 ± 1.45[b]	73.52 ± 5.99[a]
E-O	43.47 ± 0.56[b]	55.70 ± 0.47[b]	56.84 ± 0.89[ab]	75.08 ± 1.34[b]
E-C	43.47 ± 8.11[b]	42.91 ± 7.38[ab]	61.59 ± 1.04[ab]	65.66 ± 9.12[ab]
O-C	43.47 ± 1.07[b]	41.99 ± 3.62[a]	57.46 ± 2.71[a]	70.46 ± 2.85[ab]

Results are mentioned as arithmetic mean ± standard deviation of three replicates. The values followed by the same letters in the same raw are not significantly different at the significance level of $p \leq 0.05$ by the Tukey test.

Disclaimer/Publisher's Note: The statements, opinions and data contained in all publications are solely those of the individual author(s) and contributor(s) and not of MDPI and/or the editor(s). MDPI and/or the editor(s) disclaim responsibility for any injury to people or property resulting from any ideas, methods, instructions or products referred to in the content.
